# Testing some psychometric properties of the Italian version of the Skin Cancer Index: A questionnaire for measuring quality of life in patients with non-melanoma skin cancer

**DOI:** 10.3389/fpsyg.2022.991080

**Published:** 2022-11-22

**Authors:** Tonia Samela, Giulia Raimondi, Francesca Sampogna, Francesco Ricci, Luca Fania, Simona Mastroeni, Roberta Fusari, Nidia Melo Salcedo, Anna Dattolo, Elena Sofia Papanikolaou, Damiano Abeni

**Affiliations:** ^1^Clinical Epidemiology Unit, IDI-IRCCS, Rome, Italy; ^2^Clinical Psychology Unit, IDI-IRCCS, Rome, Italy; ^3^Department of Human Science, European University of Rome, Rome, Italy; ^4^Dermatology Unit, IDI-IRCSS, Rome, Italy

**Keywords:** keratinocytes, outcome measure (healthcare), psychodermatology, skin disease, italian preliminary validation

## Abstract

**Objectives:**

Non-melanoma skin cancers (NMSC) include two main types: basal cell carcinoma (BCC) and cutaneous squamous cell carcinoma (cSCC). Generic Health-Related Quality of Life (HRQoL) instruments revealed little to no HRQoL impairment in NMSC patients. Instead, the use of specific skin disease HRQoL tools contradicted those observations. For example, the Skin Cancer Index (SCI) was suggested as a validated instrument for the evaluation of the impact of skin cancers on HRQoL, and has already been validated in several languages, but not in Italian. The aim of this study is to testing some psychometric properties of the Italian version of the SCI questionnaire in a large sample of NMSC patients.

**Methods:**

This is a cross-sectional, single-center, observational study. Firstly, different factor models proposed in the literature were compared and the model with the best fit was identified. Secondly, the psychometric properties of the SCI, convergent validity and reliability, were evaluated.

**Results:**

The sample was composed of 371 NMSC patients. The factor analysis revealed that a revised version of the original model had the best fit [χ^2^(df = 85) = 354.53, *p* < 0.001, RMSEA = 0.09, CFI = 0.98, TLI = 0.97, SRMR = 0.03]. The SCI had satisfactory internal consistency for all subscales (Emotional subscale: ordinal alpha = 0.95; Social subscale: ordinal alpha = 0.94; Appearance subscale: ordinal alpha = 0.94). The convergent validity with Skindex-17 psychosocial subscale was adequate for all the SCI subscales (Emotional Subscale: rho = −0.50; Social Subscale: rho = −0.54; Appearance subscale: rho = −0.44; Total Skin Cancer Index: rho = −0.56; and *p* < 0.001).

**Conclusion:**

The tested psychometric properties of the Italian version of the SCI may suggest that it is an appropriate tool to measure the HRQoL in NMSC patients, however, further studies are needed in order to confirm and tested other psychometric features of this tool.

## Introduction

Non-melanoma skin cancers (NMSC) include two main types: basal cell carcinoma (BCC) and cutaneous squamous cell carcinoma (cSCC). Their incidence has been estimated to be 18–20 times higher than that of melanoma (Fania et al., 2021b). NMSC are an important health issue worldwide due to the aging population and the widespread sun exposure (Fania et al., 2020, 2021a). Even if the mortality from BCC is negligible, and even if the cSCC (whether *in situ* or as an invasive form) prognosis is good (with a 5-year survival ≥90%), these cancers can be associated with relevant morbidity (Miller and Weinstock, 1994; Skulsky et al., 2017) and a significant impact on people’s health related quality of life (HRQoL) (Chernyshov et al., 2015; Alam et al., 2018). Furthermore, recurrences and subsequent NMSC lesions occur often (Trakatelli et al., 2007) and temporary or permanent scars caused by treatment sequelae could be associated with functional and esthetic issues (Kauvar et al., 2015) that may considerably affect HRQoL (van der Geer et al., 2010).

Even though one of the first published studies addressing the effect of BCC on HRQoL demonstrated little impact with only minimal differences before and after treatment (Blackford et al., 1996), and a review by Gibbons et al. (2013) concluded that generic HRQoL instruments revealed little to no HRQoL impairment in NMSC patients (Gibbons et al., 2013), the use of specific skin disease HRQoL tools contradicted those observations.

As it is stressed by Sampogna et al. (2019), comparing HRQoL of melanoma and NMSC patients using Skindex-17, the symptomatic component of HRQoL was more affected in patients with NMSC than in those with melanoma (Sampogna et al., 2019), and even the impact on the psychosocial component was not negligible (Gaulin et al., 2015). Moreover, the Skindex (both in its extended version and in its 17-item version) is a widely recognized tool that has been used for many years to measure the quality of life of people with different skin disorders (Sampogna et al., 2012), has good psychometric properties and is adequately sensitive and reliable (Nijsten et al., 2006). In addition, this instrument contains a total score and a subscale score called “psychosocial subscale” that is well suited to comparison with SCI. For these reason, among others, we have chosen this tool for comparison.

The Skindex-17 and SCI both measure constructs such as psychological and social functioning, but only SCI contains three specific items related to patients’ appearance perception. Both questionnaires included a total HRQoL scale, so we hypothesized that the SCI and Skindex-17 would be highly correlated, but not overlapping, in their general HRQoL measurement.

As stated in a position paper of the European Academy of Dermatology and Venereology Task Force on Quality of Life (Chernyshov et al., 2019), the generic or even the dermatology-specific instruments may not be sensitive enough to measure the total impact of skin cancer on patient’s lives. The Skin Cancer Index (SCI; Rhee et al., 2006) was suggested as a validated instrument for the assessment of the impact of skin cancer on HRQoL, and has already been validated in several languages (i.e., English by Rhee et al., 2006; in Spanish by De Troya-Martín et al., 2015; and Canadian French by Moran et al., 2021).

A tool assessing HRQoL of NMSC patients is also needed for the Italian population, given their high life expectancy, the frequent occupational and leisure-time UV exposure, and the ever-increasing intensity of UV radiation. Therefore, the aim of the present study was to validate some psychometric properties of the Italian version of the SCI questionnaire in a large sample of NMSC patients. Specifically, the first aim was to compare different factor models proposed in the literature (Rhee et al., 2006; De Troya-Martín et al., 2015; Moran et al., 2021) and to identify the model with the best fit. The second aim was to investigate the convergent validity, and reliability of the SCI.

## Materials and methods

### Setting, study design, and sample

This is a cross-sectional, single-center, observational study. The research has been conducted in accordance with the Declaration of Helsinki (64th WMA General Assembly, Fortaleza, Brazil, October 2013) and was approved by the Institutional Ethical Committee (protocol number n°608/1, 17th September 2019). All studies are registered by the Institutional Ethical Committee, which in turn, every year, transfers relevant information (e.g., enrolment open or close, number of enrolled patients during the past year, etc.) to the Italian Ministry of Health.

Data were collected from October 10, 2019 to December 14, 2021 at the dedicated NMSC outpatients clinic of the dermatological research hospital IDI-IRCCS, Rome, Italy.

Being a registry-based study, no sample size or power calculation was performed, however, we have reached an adequate number of participants, considering a sample size of at least 50 patients adequate for the assessment as asserted by Terwee et al. (2007). Consecutive patients were enrolled within the framework of the IDI-IRCCS NMSC Registry. Included patients had to satisfy the following inclusion criteria: (i) history or clinical suspicion of NMSC diagnosed by expert dermatologists assigned to the specific NMSC outpatients clinic and following NCCN guidelines for NMSC (NCCN Guidelines® for Basal Cell Skin Cancer, Version. 1.2022, 2021; Schmults et al., 2021); (ii) a good fluency of spoken and written Italian, and (iii) the obtainment of the written informed consent, signed by the patient if 18-and-over years old or by a parent/guardian for patients younger than 18 years of age. Exclusion criteria were: (i) any diagnosed major psychiatric disorders or cognitive impairment.

### Data collection procedures

Data collected included demographic and clinical information. Demographic variables included sex, age, weight, and height in order to calculate Body Mass Index (BMI), educational level, marital status, and job status (employment), alcohol and tobacco intake, and number of hours of sun exposure. Clinical variables included type of NMSC, family history of cancer, patient global assessment (PtGA), and physician global assessment (PGA). The PGA was ranked for each participant by the dermatologist during the medical examination. It is a frequently used measure to assess clinical severity in dermatology (Pascoe et al., 2015). The SCI and Skindex-17 questionnaires were handed out to the patients who agreed to complete them. The Skindex-17 (Nijsten et al., 2006) is a dermatological self-report HRQoL instrument composed by 17 items. Answers are given on a three-point Likert scale (i.e., “never” to “often/always”). It measures the burden of dermatologic conditions on two scales: psychosocial and symptoms. Higher scores correspond to a worst condition experienced by patients. In this sample the total and Skindex-17 subscale scores had an adequate reliability (i.e., Skindex-17 total score Cronbach alpha = 0.93; Symptoms subscale score: α = 0.82; Psychosocial subscale score: α = 0.92).

The research questionnaires and the clinical case report forms are then processed by trained personnel in the clinical epidemiology unit (i.e., NMS, SM, and RF) who perform data management, data entry, and quality control.

### The skin cancer index

The skin cancer index (SCI; Rhee et al., 2007) is a 15-item self-report measure that so far has demonstrated the best evidence of its usefulness in patients with BCC and SCC (Lee et al., 2013). The SCI assesses HRQoL on three subscales: Emotional Subscale (i.e., anxiety, worry, and frustration), Social Subscale (i.e., meeting new people and going out in public), and Appearance Subscale (i.e., worry about dimension and noticeability of the scars). The scales are constituted by seven, four, and four items, respectively. Each item is rated on a five-point Likert scale, and the raw scores are transformed from a 0 (worst) to 100 (best) standardized score. Subscales total scores are obtained by summing the standardized scores and dividing by the number of items of each subscale. The total Skin Cancer Index score is computed by summing the subscale total scores and dividing by 3 (Rhee et al., 2006).

Differently from the original validation study (Rhee et al., 2006), De Troya-Martín et al. (2015) found evidence for a two-factor structure, combining the social and appearance subscales, which reported satisfactory Cronbach’s alpha (i.e., both of 0.87), with 12 items (items #2, #9, and #13 were removed because of low factor loadings). Also Moran et al. (2021) reported results for a two-factor structure, with no item removed, however, the model did not fit the data well [χ^2^(df = 89) = 326.44, *p* < 0.001, RMSEA = 0.12, 90% CI [0.11, 0.14], CFI = 0.96, TLI = 0.96]. Also, a one-factor model and a hierarchical model were tested, but both these models did not fit the data (Moran et al., 2021).

### Study phases

To adapt and validate the original SCI scale, a study with two consecutive phases was designed. The first phase, summarized in Table 1, referred to the Italian translation and adaptation of the SCI, and was carried out according to Beaton’s guidelines (Beaton et al., 2000). The second phase referred to the evaluation of the psychometric properties of the questionnaire. To assess the questionnaire’s factorial structure and reliability, exploratory factor analysis, and ordinal alpha were conducted, respectively. To indicate the convergent validity (i.e., the measurement of theoretically similar constructs that should be highly inter-correlated, that can be estimated using correlation coefficients), Spearman’s correlation analysis was performed with the Skindex-17 for which reliability and validity studies have been conducted in the Italian-speaking population (Nijsten et al., 2006).

**Table 1:** SCI process of translation and adaptation.

**1. Forward translation**	Three authors (DA and FS—epidemiologists—and FR—dermatologist), with vast experience in the terminology of the area covered by the tool and with interview skills, took part in the project, and provided three independent versions. The translators are knowledgeable of the English-speaking culture, but their mother tongue is Italian. Conceptual equivalents of a word or phrase, not a literal translation, were seeked—triving to be simple, clear, and concise. The most common language, considering the typical respondent, was used trying to avoid jargon, colloquialism, or offensive term. The three translators then sat down together and produced a common version, based on consensus, by blending the best solutions found in the three independent translations.
**2. Expert panel Back-translation**	Using the same approach used in first step, the instrument was then translated back to English by an independent mother-tongue translator.
**3. Review committee**	The final version was submitted to a bilingual committee consisting of clinicians and translators. The text was checked for semantic and idiomatic equivalence acceptable for dynamic equivalence. The committee reached a consensus on any discrepancy. Step 3 ended with a final approval.
**4. Pre-testing and cognitive interviewing**	A provisional version of the Italian SCI was pre-tested on 30 patients during their follow-up clinic visit. Patients accepted to complete the questionnaire and then they were interviewed by the clinical psychologists. Subjects were asked about the degree of difficulty encountered when answering the questionnaire and to comment on the comprehensiveness and clarity of the items in the SCI.
**5. Final version**	The final version of the Italian adaptation of SCI was the result of all the iterations described above (Figure 2).

### Statistical analysis

All the analyses were performed with Mplus 8.3 (Los Angeles, CA: Muthén & Muthén; Muthén and Muthén, 2017), R Core Team (2021), and the Statistical Package for the Social Sciences (SPSS, 2013), and G power (Faul et al., 2007).

All patients who reported missing data in SCI and/or Skindex-17 questionnaire were excluded from the analyses. Instead, it was not possible to achieve the same in the socio-demographic variables; indeed, the exact frequencies of the available data are shown in Table 2. For the purpose of describing sociodemographic and clinical features of the sample, descriptive statistics were obtained through the computation of mean and standard deviation (SD) for the quantitative variables and through the frequency distribution for the qualitative ones.

**Table 2:** Sociodemographic and clinical features of the sample.

Variables	Levels	*N* ^*^	%	mean	SD
*Overall*	*--*	371	100.0		
Sex	*f*	171	46.1		
	*m*	200	53.9		
NMSC type	BCC(+AK)	162	46.4		
	SCC(+AK)	117	33.5		
	BCC + SCC	46	13.2		
	Other	24	6.9		
Age (years)				67.8	±12.2
BMI kg/m2				25.1	±3.8
Education	None/elementary school	30	8.5		
	Middle school	72	20.6		
	High school	156	44.6		
	College degree or higher	92	26.3		
Marital status	Single	48	13.9		
	Married	244	70.5		
	Widow/er	29	8.4		
	Divorced	25	7.2		
Alcohol intake	None	184	53.0		
	Yes	60	17.3		
	Occasionally	103	29.7		
Smoking	None	193	54.4		
	Yes	53	14.9		
	Ex-smoker	109	30.7		
Family history of cancer	None	128	36.6		
	Yes	74	63.4		
Sun exposure	Hours per working day	301		3.1	±2.5
	Hours per non-working day	302		4.3	±3.3
PGA		157		2.11	±0.85
PtGA		207		2.54	±1.02

^*^Totals may vary due to missing data. SD, standard deviation; NMSC, non-melanoma skin cancer; BCC, basal cell carcinoma; SCC, squamous cell carcinoma; AK, actinic keratosis; m, males; f, females; BMI, body mass index; PtGA, patient global assessment; and PGA, physician global assessment.

To test whether the data were suitable for factor analysis, the Bartlett’s test of sphericity and the Kaiser–Meyer–Olkin (KMO) test were performed. Adequacy of the correlation matrix is suggested by a significant Bartlett’s test (*p* < 0.05) and a KMO index >0.70. Multiple Confirmatory Factor Analyses (CFAs) testing the different models proposed in the literature were conducted using a mean-and variance-adjusted weighted least square (WLSMV) estimator, given the categorical nature of the variables, with a polychoric correlation matrix. To perform a factor analysis a large sample size is required. The usually recommended minimum number of individuals is 200 (Kyriazos, 2018). Models fit were evaluated using the following indices: (1) the root mean square error of approximation (RMSEA), with values below 0.05 indicating evidence of absolute fit, values between 0.05 and 0.09 indicating the adequacy of the model, and values above or equal to 0.10 indicating the poor fit of the model (Browne and Cudeck, 1992; Hu and Bentler, 1999); (2) the Tucker–Lewis Index (TLI), with values >0.95 indicating the good fit of the model and values of 0.90 and higher an acceptable fit; (3) the Comparative Fit Index (CFI), with values >0.95 indicating good model fit and values of 0.90 and higher an acceptable fit; (4) the standardized root mean square residual (SRMR), with values <0.08 indicating good fit (Yu, 2002); and (5) the chi-square (χ2) test, with *p* values greater than 0.05 indicating an adequate fit to the data. However, χ^2^ is sensitive to sample size, and so *p* values might become significant for large samples (Schumacker and Lomax, 2012). In case of suboptimal fit of all the models tested, a parallel analysis (PAs; Horn, 1965) was performed to determine the number of factors to retain.

Eventually, if the model suggested in PA did not report a satisfactory fit, large modification indexes (>10) of the model with the most acceptable fit were inspected to look for refinements to add to the model. Modification indices (MIs) may suggest the need to add a path between variables or constraint/free one or more parameters. Values greater than 10 indicate that the model would be improved if a MI is applied, and the *p* value for the new parameter would be <0.001. However, since refinement of the factor model based on MIs is a data driven approach, modification indices should be considered one by one, and the model should be tested each time. A final CFA, based on the results from the MIs was performed, and model fit was evaluated with the same indices reported above.

Moreover, indices of internal consistency [i.e., ordinal alpha, the Molenaar Sijtsma statistic (MS), and latent class reliability coefficient (LCRC)] of the SCI were calculated. Ordinal alpha was used as a reliability estimations method, rather than Cronbach’s alpha, because the SCI is an ordinal type scale, which cannot be considered as continuous (Gadermann et al., 2012).

Convergent validity of SCI was assessed with Skindex-17 by examining the correlation between individual scores in SCI and in Skindex-17 subscales and total score. Factor scores of each SCI subscales were calculated and used to compute the correlation analyses. Finally, to assess the adequacy of our sample size for convergent validity, a G Power analysis (Faul et al., 2007) was conducted. A minimum of 64 participants was required to provide adequate statistical power (1-β = 80%) in order to detect a moderate effect size (ES = 0.30) with α =0.05.

## Results

Three hundred and seventy-one patients diagnosed with NMSC, 53.90% males, with a mean age of 67.80 (±12.25) years, completed all questionnaires without any missing data. Mean PGA and PtGA scores for the present sample were attested at 2.11 (±0.85) and 2.54 (±1.02) respectively. We compared the patients included in this study (i.e., patients with complete answers to the SCI and the Skindex-17) with those who are in the registry but were excluded because of missing values, and we found no differences in age (*t*-test = 1.408, *p* = 0.993), gender [χ2(df = 1) = 0.799, *p* = 0.371], Physician Global Assessment (*t*-test = 1.439, *p* = 0.917), and Patient Global Assessment (*t*-test = 0.679, *p* = 0.408). Demographic and clinical data related to the patients are reported in Table 2.Totals of some variables in Table 2 may vary due to missing data (i.e., 22 missing for “NMSC type”; 21 missing data for “Education”; 25 missing item for “Marital status”; 24 missing data for “Alcohol intake”; 16 missing data for “Smoking”; 169 missing data for “Family history of cancer”; 214 missing data for “PGA”; and 164 missing data for “PtGA”; Table 2).

The correlation matrix was adequate for factor analysis (Bartlett’s test of sphericity = 4640.05; df = 105; *p* < 0.001; KMO = 0.93). Table 3 reports the fit of the competing models tested. The CFAs of the models proposed in the literature (Model-1, Model-2, and Model-3) did not fit the data well, while Model-4 was not identified. For all the models tested, all items reported high factor loadings (≥ 0.71), which did not identify the problematic items. Since the CFA of the original model indicated a high correlation between the Emotional and Appearance factors (*r* = 0.90), a two-factor model combining these two factors was tested (Model-5). However, it did not show a good fit. The PA suggested the presence of three factors, however, since only the third factor had an eigenvalue <1 (= 0.21) and reported low factor loadings (< 0.35) for all items, only a two-factor solution (Model-6) was retained, but it still did not yield a good fit.

**Table 3:** Fit results of the competing models tested (*N* 371).

	Model description	χ^2^ (*df*)	RMSEA (95%CI)	CFI TLI	SRMR
**Model-1** (Rhee et al., 2006)	Original model with three factors and 15 items	555.86 (87)	0.12 (0.11–0.13)	0.97 0.96	0.05
**Model-2** (De Troya-Martín et al., 2015)	Two factors and 12 items (items #2, #9, and #13 excluded)	452.78 (53)	0.14 (0.13–0.15)	0.97 0.96	0.06
**Model-3** (Moran et al., 2021)	Two factors and 15 items	660.68 (89)	0.13 (0.12–0.14)	0.96 0.95	0.06
**Model-4** (Moran et al., 2021)	1 second-order factor, two first-order factors, and 15 items	Model not identified			
**Model-5**	Two-factor model (due to high correlation of the Emotional and Appearance factors in the original model)	621.77 (89)	0.12 (0.11–0.13)	0.96 0.96	0.05
**Model-6**	Two-factor model derived from the PA	119.98 (76)	0.11 (0.10–0.12)	−0.88	0.04
**Model-7** (Refined model)	Original model with two cross-loading items as suggested by M.I.	354.53 (85)	0.09 (0.08–0.10)	0.98 0.98	0.03

χ^2^, chi squared; *df*, degrees of freedom; RMSEA, Root Mean Square Error of Approximation; CFI, comparative fit index; TLI, Tucker Lewis Index; SRMR, Standardized Root Mean Squared Residual; PA, parallel analysis; MI, modification indices; “-,”values were not reported in the output.

**Figure 1:**
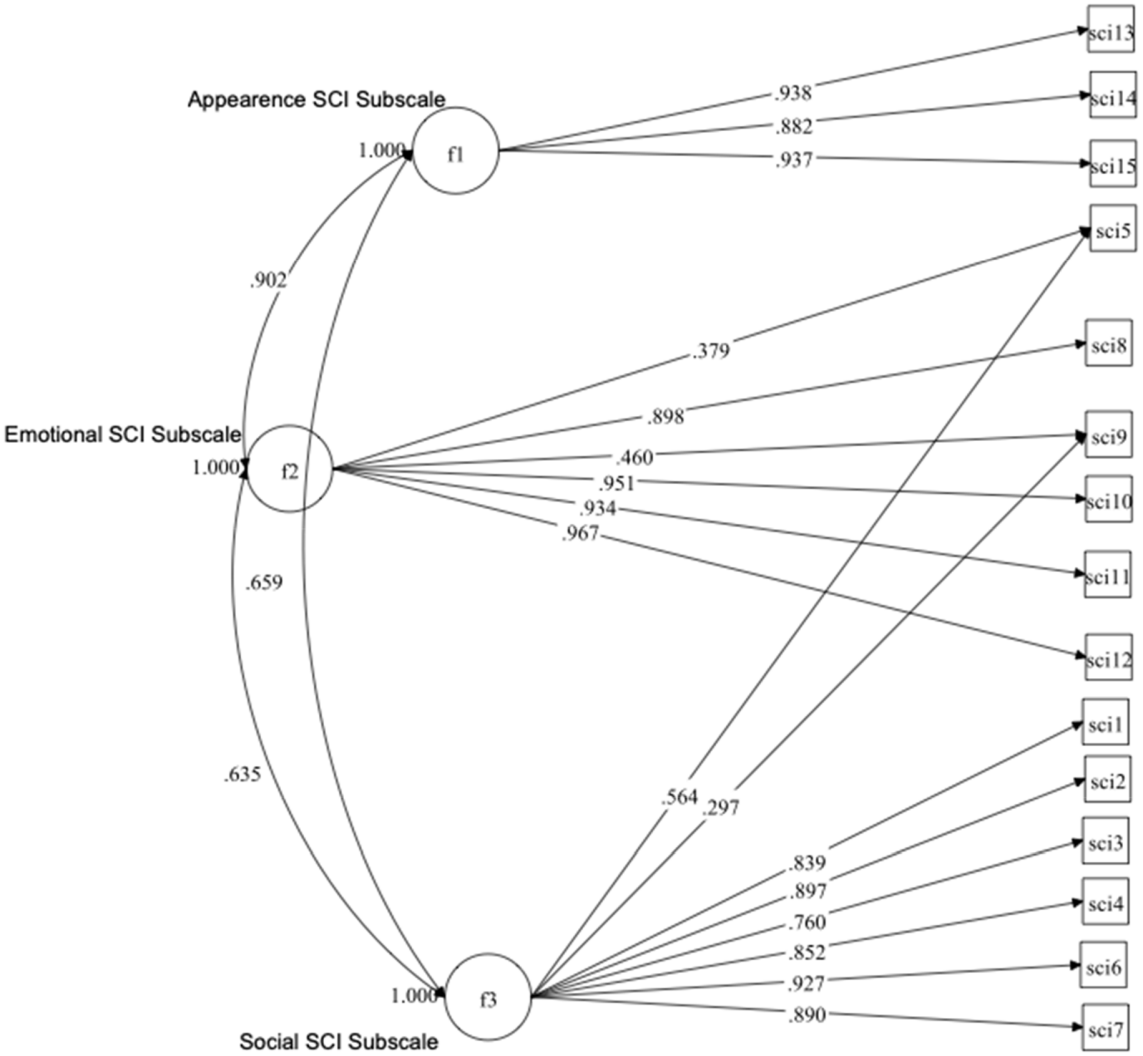
Graphical representation of the refined model (Model-7), with standardized values.

Since the original model (Model-1) showed the most acceptable fit, the MI of this model was looked-for. MIs suggested the cross-loadings of two items (item #5 and #9), which were associated with the highest estimated parameter change (0.49 and 0.50, respectively), and significant (*p* < 0.001) changes in the value of the χ^2^ statistic. Specifically, item #5, which belongs to the Social subscale, was suggested to cross-load in the Emotional subscale, and item #9, which belongs to the Emotional subscale, was suggested to cross-load in the Social subscale. The decision to add these two cross-loadings was also supported theoretically, since the meaning of these items fits both the Emotional and Social subscales (item #5, “*Felt frustrated about your skin cancer?*” and #9, “*Felt concerned that your skin cancer may worry friends or family?*”). The refined model (Model-7) had an acceptable fit to the data [χ^2^(df = 85) = 354.53, *p* < 0.001, RMSEA = 0.09, CFI = 0.98, TLI = 0.97, SRMR = 0.03; Figure 1].

**Figure 2:**
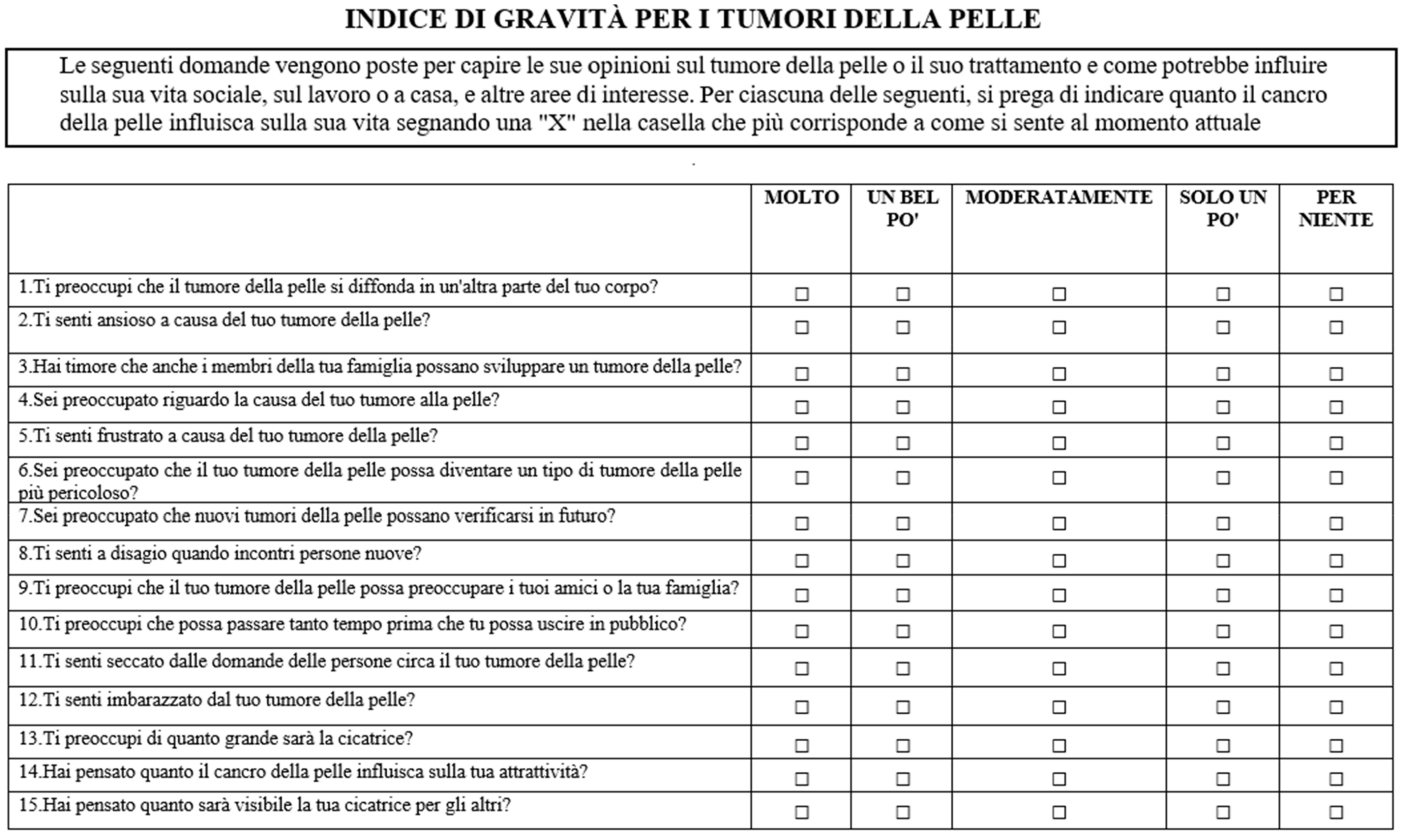
SCI Italian translation.

The whole SCI scale had satisfactory internal consistency (ordinal alpha = 0.96, MS = 0.94, and LCRC = 0.95), as well as each subscale (Emotional: ordinal alpha = 0.95, MS = 0.91, and LCRC = 0.92; Social: ordinal alpha = 0.94, MS = 0.92, and LCRC = 0.92; Appearance: ordinal alpha = 0.94, MS = 0.90, and LCRC = 0.91). It is relevant to know that the reliability of each subscale was computed by including the fifth item in the Emotional subscale, and the ninth item in the Social subscale.

In Table 4, we reported the Spearman correlation coefficients between SCI subscales, SCI Total Skin Cancer Index, and Skindex-17 subscales. The scores of the Skindex-17 and SCI subscales (i.e., SCI Emotional subscale; SCI Social subscale; SCI Appearance subscale; Total Skin Cancer Index; Skindex-17 Psychosocial subscale; and Skindex-17 Symptoms subscale) were satisfactorily and significantly related to each other (*p* < 0.001).

**Table 4:** Spearman correlation between SCI subscales, SCI Total Skin Cancer Index, and Skindex-17 subscales (N 371).

	SCI Social subscale	SCI Appearance subscale	Total Skin Cancer Index	Skindex-17 Psychosocial subscale	Skindex-17 Symptoms subscale
SCI Emotional subscale	0.70^*^	0.56^*^	0.91^*^	−0.50^*^	−0.38^*^
SCI Social subscale		0.70^*^	0.88^*^	−0.54^*^	−0.44^*^
SCI Appearance subscale			0.78^*^	−0.44^*^	−0.29^*^
Total Skin Cancer Index				−0.56^*^	−0.43^*^
Skindex-17 Psychosocial subscale					0.68^*^

Negative correlation coefficients depend on the scoring of the instruments: in SCI higher scores indicate a better quality of life, while in Skindex-17 higher scores indicate a worse quality of life. ^*^p < 0.001.

## Discussion

This paper describes the adaptation and validation of some psychometric properties of the Italian version of the SCI, with the intent to achieve a linguistic and semantic equivalence with the original questionnaire proposed in English (Rhee et al., 2006). Our results supported the model originally proposed by Rhee et al., (2006), with three factors (corresponding to Appearance, Emotion, and Social subscales) and 15 items. As in the original instrument, our analysis identified two separate subscales for the evaluation of appearance and emotions. In fact, the first construct refers to worry about physical appearance, while the second focuses on worry, anxiety, and frustration due to the clinical characteristics of the condition. However, in the present study, we had to revise the original three-factor model by including two cross-loadings for items #5 and #9, as suggested by the MI. Specifically, item #5 (“*Felt frustrated about your skin cancer?*”), which loaded on the social subscale in the original model, was also loaded on the emotional subscale and item #9 (“*Felt concerned that your skin cancer may worry friends or family?*”), which loaded on the emotional subscale in the original model, was also loaded on the social subscale. This decision was also supported by considering the meaning of these items, which seem to assess both the social and emotional aspects at the same time. Perhaps this result was not found in other studies because of cultural differences, which can also explain why other studies reported findings for different numbers of factors and also had to remove some items. On the basis of this interpretation, we have decided to include the two cross-loadings to compute the subscales scores by using the factor scores. Therefore, both item #5 and item #9 were used to compute both Social and Emotional subscales scores. Moreover, these cross-loadings items seem to explain different aspects of each subscale, which would be missing if we did not include them to compute the subscales scores. Finally, the SCI index, as well as each subscale, showed satisfactory internal consistency.

This Italian adaptation was created following the current recommendations for the cultural adaptation and validation of patient-reported outcome measures (PROMs; Guillemin et al., 1993; Herdman et al., 1998; Beaton et al., 2000). As concerns manageability, the Italian adaptation of the SCI achieved a high degree of acceptability and patients did not require assistance to complete the questionnaire, managing to respond to all items in a self-administered way, through a Likert-scale evaluation. Moreover, the total and subscale scores are easy to obtain (i.e., summing each item score), and then it is possible to convert the total score to a scale from 0 to 100. None of these procedures require specific software.

Concerning field practice, SCI could be a particularly specific and useful tool that allows clinicians and researchers to analyze the psychological and emotional impact of NMSC on patients’ lives. This information allow to operationalize and compare the psychological impact of NMSC in the evaluation of potential therapeutic alternatives to invasive surgery for BCC. On the other side, SCI could help clinicians to recognize patients who need a more in-depth psychological assessment, in order to provide a specific psycho-oncological counseling to patients and/or their caregivers. From this perspective the SCI could also be considered as a screening tool useful to investigate the psychological well-being of patients suffering from NMSC.

The Italian version of the SCI showed good convergent validity with the Skindex-17, especially for the psychosocial scale.

As hypothesized, the SCI and the Skindex-17, despite being correlated, were not overlapping. This result confirms that dermatology-specific and NMSC-specific questionnaires do not measure the same aspects of HRQoL impairment. As stated in the position paper by Chernyshov et al. (2019), the dermatological component may not be the main source of problems in patients with skin cancer. Especially at a late stage of the disease, the psychological burden may overcome the skin problems and thus cancer-specific or disease-specific questionnaires may be more appropriate. The items of the SCI are particularly focused on worry, which is a component of anxiety and a frequent emotion in people with cancer in general, and in particular in NMSC (Khoshab et al., 2020). In fact, it has been observed that even after surgery, patients with facial NMSC report cancer worry (van Hensbergen et al., 2022), and such emotion has a strong impact on patients’ quality of life and wellbeing (Radiotis et al., 2014). In this sample, the SCI had satisfactory internal consistency for all subscales. For this reason, it could be possible to assert that SCI has a good possibility in proving that the different test-items who belong to the same construct produce similar results. So, the three constructs assessed by SCI are consistent and related but only partially overlapping. From this evidence, it is possible to hypothesize that a low level of self-appearance acceptance could have repercussions in psychological or social well-being, or it may also be possible that people with better levels of social and psychological life could better accommodate appearance issues resulting from NMSC sequelae.

The current study and the Italian version of the SCI have various strengths. As for the study: (i) we used an up-to-date statistical approach which also considered the categorical nature of the variables analyzed; (ii) we were also able to investigate the validity of the SCI; (iii) this Italian adaptation has demonstrated a high structural similarity with the original model (Rhee et al., 2006); and (iv) the women/men ratio in our sample was well balanced.

However, some limitations of this study affecting the generalizability of the results should also be noted. First, we did not investigate the stability of the factor structure of the SCI over time. Second, given the cross-sectional nature of the data, test–retest validity could not be assessed. Future studies will be needed to investigate and confirm our results on the factorial structure of SCI. Moreover, multi-group CFA for measurement invariance of the SCI are needed to compare the results across different language versions of the questionnaire and cultures. In the current study, we were not able to perform a measurement of invariance analysis due to not adequate sample size (< 200 subjects for the female group; Meade, 2005). Third, the presence of cross-loadings suggests that the SCI needs to be extensively revised. This is also supported by the fact that the results of the PA did not match the ones reported by the CFA (Li et al., 2020) and, also, by the fact that the other studies that tried to validate the SCI in other languages reported different findings in matter of its factor structure.

Finally, it was not possible to quantitatively depict the clinical or demographic features of participants who refused to took part to the research, because no systematic data collection was performed. This could be a limit in considering the tool manageability. However, from a qualitative point of view, the reasons that led the participants to refuse were: advanced age, contextual or chronic sensory, and/or motor deficits (due to the advanced age, a typical feature of the population affected by NMSC), and difficulties in getting space and time that are due to the setting in which the study was carried out.

In conclusion, the SCI could be a promising tool for assessing the quality of life, self-acceptance, social and psychological problems of people with NMSC, also in the Italian context. However, other research is needed to test in different contexts the features of this tool.

## Data availability statement

The raw data supporting the conclusions of this article will be made available by the authors, without undue reservation.

## Ethics statement

The studies involving human participants were reviewed and approved by Istituto Dermopatico dell’Immacolata Ethical Committee, IDI-IRCCS. The patients/participants provided their written informed consent to participate in this study.

## Author contributions

TS, FR, FS, and DA: conceptualization. SM, RF, NMS, AD, EP, TS, and LF: enrollment and data curation. GR, TS, and DA: methodology and formal analysis. FS, FR, and DA: project administration and supervision.^.^TS and GR: writing—original draft. All authors contributed to the article and approved the submitted version.

## Funding

This study was supported in part by “Progetto Ricerca Corrente 2021–2022” of the Italian Ministry of Health.

## Conflict of interest

The authors declare that the research was conducted in the absence of any commercial or financial relationships that could be construed as a potential conflict of interest.

## Publisher’s note

All claims expressed in this article are solely those of the authors and do not necessarily represent those of their affiliated organizations, or those of the publisher, the editors and the reviewers. Any product that may be evaluated in this article, or claim that may be made by its manufacturer, is not guaranteed or endorsed by the publisher.
